# Inhibition of EP4 Signaling Attenuates Aortic Aneurysm Formation

**DOI:** 10.1371/journal.pone.0036724

**Published:** 2012-05-03

**Authors:** Utako Yokoyama, Ryo Ishiwata, Mei-Hua Jin, Yuko Kato, Orie Suzuki, Huiling Jin, Yasuhiro Ichikawa, Syun Kumagaya, Yuzo Katayama, Takayuki Fujita, Satoshi Okumura, Motohiko Sato, Yukihiko Sugimoto, Hiroki Aoki, Shinichi Suzuki, Munetaka Masuda, Susumu Minamisawa, Yoshihiro Ishikawa

**Affiliations:** 1 Cardiovascular Research Institute, Yokohama City University, Yokohama, Japan; 2 Department of Surgery, Yokohama City University, Yokohama, Japan; 3 Department of Pharmaceutical Biochemistry, Kumamoto University, Kumamoto, Japan; 4 Cardiovascular Research Institute, Kurume University, Kurume, Japan; 5 Department of Life Science and Medical Bio-Science, Waseda University Graduate School of Advanced Science and Engineering, Tokyo, Japan; University of Tokyo, Japan

## Abstract

**Background:**

Aortic aneurysm is a common but life-threatening disease among the elderly, for which no effective medical therapy is currently available. Activation of prostaglandin E_2_ (PGE_2_) is known to increase the expression of matrix metalloproteinase (MMP) and the release of inflammatory cytokines, and may thus exacerbate abdominal aortic aneurism (AAA) formation. We hypothesized that selective blocking of PGE_2_, in particular, EP4 prostanoid receptor signaling, would attenuate the development of AAA.

**Methods and Findings:**

Immunohistochemical analysis of human AAA tissues demonstrated that EP4 expression was greater in AAA areas than that in non-diseased areas. Interestingly, EP4 expression was proportional to the degree of elastic fiber degradation. In cultured human aortic smooth muscle cells (ASMCs), PGE_2_ stimulation increased EP4 protein expression (1.4±0.08-fold), and EP4 stimulation with ONO-AE1-329 increased MMP-2 activity and interleukin-6 (IL-6) production (1.4±0.03- and 1.7±0.14-fold, respectively, *P*<0.05). Accordingly, we examined the effect of EP4 inhibition in an ApoE^−/−^ mouse model of AAA infused with angiotensin II. Oral administration of ONO-AE3-208 (0.01–0.5 mg/kg/day), an EP4 antagonist, for 4 weeks significantly decreased the formation of AAA (45–87% reduction, *P*<0.05). Similarly, EP4^+/−^/ApoE^−/−^ mice exhibited significantly less AAA formation than EP4^+/+^/ApoE^−/−^ mice (76% reduction, *P*<0.01). AAA formation induced by periaortic CaCl_2_ application was also reduced in EP4^+/−^ mice compared with wild-type mice (73% reduction, *P*<0.001). Furthermore, in human AAA tissue organ cultures containing SMCs and macrophages, doses of the EP4 antagonist at 10–100 nM decreased MMP-2 activation and IL-6 production (0.6±0.06- and 0.7±0.06-fold, respectively, *P*<0.05) without increasing MMP-9 activity or MCP-1 secretion. Thus, either pharmacological or genetic EP4 inhibition attenuated AAA formation in multiple mouse and human models by lowering MMP activity and cytokine release.

**Conclusion:**

An EP4 antagonist that prevents the activation of MMP and thereby inhibits the degradation of aortic elastic fiber may serve as a new strategy for medical treatment of AAA.

## Introduction

Aortic aneurysm is the 13^th^ leading cause of death in the United States, with roughly 15,000 deaths per year [1]. After rupture occurs, the probability of mortality is greater than 60% [1]. Ultrasonography screening studies of men over 60 years old have shown that a small abdominal aortic aneurysm (AAA), i.e., 3 to 5 cm in diameter, is present in 4% to 5% of patients [2], [3]. When patients with a small AAA were followed for up to 6 years, AAA diameter had increased in 55% of patients. The rate of increase in diameter was more than 1 cm per year in 23% of patients, and AAA diameter had expanded to 6 cm in 9% of patients, at which point the risk of rupture significantly increases [3]. Although AAAs typically continue to expand, increasing the likelihood of rupture and consequent mortality, no effective pharmacological therapy to prevent the progression of AAA is currently available.

The hallmarks of AAA are the presence of an inflammatory infiltrate within the vascular wall, which is followed by proteolytic degradation of extracellular matrixes (ECM) [4]. Proinflammatory cytokines play an important role, particularly in the initiation of aneurysms [1]. Inflammatory mediators such as interleukin-6 (IL-6), IL-1β and monocyte chemoattractant protein-1 (MCP-1) are released in the AAA wall [5], [6]. In an experimental AAA model of ApoE^−/−^ mice infused with angiotensin II (AngII), IL-6 and MCP-1 production were both increased [7]. In contrast, the incidence of AAA was decreased after AngII infusion in mice lacking either the IL-6 or MCP-1 receptor CCR2 [7]. Proteolytic enzymes, together with inflammatory mediators, promote extensive structural remodeling of the arterial wall, characterized by the degradation of ECM such as elastic fibers [8]. Activation of proteolytic enzymes, particularly matrix metalloproteinases-2 (MMP-2) and MMP-9 in the tunica media, is considered to be an important cause. These MMPs exacerbate aortic dilatation, as demonstrated in studies using human patients or genetically engineered mice [8], [9].

Cyclooxigenase-2 (COX-2)-dependent prostaglandin E_2_ (PGE_2_) synthesis is induced during the development of aneurysms [5], [10]. PGE_2_ synthesized by macrophages and smooth muscle cells (SMCs) increases the production of MMPs [11], [12] and stimulates the production of cytokines [5]. Selective COX-2 inhibition, as induced by celecoxib or genetic disruption of COX-2, decreased AngII-induced AAA formation in mice [13], [14]. Despite these positive findings, however, administration of selective COX-2 inhibitors has increased the frequency of adverse cardiovascular events, as reported in clinical studies [15], [16]. Nonetheless, inhibition of pathophysiologic COX-2-dependent PGE_2_ signaling may still remain an attractive therapeutic strategy.

The present study was designed to examine the hypothesis that the prostanoid receptor, which is downstream of COX-2-dependent PGE_2_ signaling, plays a critical role in the formation of AAA. We demonstrate that prostanoid receptor EP4 expression was increased in SMCs from human AAA tissue, and that EP4 stimulation enhanced MMP-2 activation and IL-6 production. Further, pharmacological inhibition or genetic disruption of EP4 signaling successfully attenuated AAA formation in mice. We also demonstrate that an EP4 antagonist attenuated MMP-2 activation and IL-6 production in the explants of human AAA.

## Materials and Methods

### Reagents

Antibody for EP4 was obtained from Cayman chemical (Ann Arbor, MI, USA). Antibodies for α-smooth muscle actin and CD68 were obtained from Sigma-Aldrich (St. Louis, MO, USA) and Dako Cytomation (Glostrup, Denmark), respectively. ONO-AE1-329 and ONO-AE3-208 were kindly provided by the ONO pharmaceutical company (Osaka, Japan).

### Human Aortic Samples

We obtained surgical specimens from individuals with AAA. We performed *ex vivo* culture using fresh AAA samples during surgery as described previously [17]. Briefly, tissues were minced to approximately 1 mm thickness, and plated on 24-well plates with 10% FBS/DMEM (Invitrogen, Carlsbad, CA, USA). Media was changed 24 h after plating. We collected some conditioned media after 48 h of incubation as a control for each well. Each well was then treated with ONO-AE1-329 or ONO-AE3-208. Conditioned media 48 h after treatment was obtained and subjected to gelatin zymography and ELISA. To compare the effect of drugs among samples, values for each well obtained from stimulated conditioned media were normalized to values from control conditioned media.

To obtain the primary culture of human aneurysm aortic smooth muscle cells (hAASMCs) from AAA tissue, the medial layer of the AAA was cut into 1- to 2-mm^3^ pieces which were placed in the explant culture on uncoated dishes in 10% FBS/DMEM (Invitrogen). Culture medium was changed after 7 days and thereafter every 3 days during a 3- to 4-week period until the specimens became confluent. The purity of the hAASMCs was confirmed by staining with α-smooth muscle actin. When confluent, SMCs were transferred (at passage 2 or 3) onto uncoated 6-well or 96-well plates for immunoblotting, gelatin zymography, and ELISA. Human aortic SMCs (hASMCs) from individuals who died of unrelated causes were obtained from Lonza (Walkersville, MD, USA).

### Cell Culture

THP-1cells were obtained from the Health Science Research Resources Bank (Osaka, Japan). We maintained hAASMCs and hASMCs in SmGM-2 containing 5% FBS and growth supplements (Lonza) and maintained THP-1 cells in RPMI1640 (Wako, Osaka, Japan) containing 10% FBS. For differentiation of THP-1 monocytes into adherent macrophages, cells were treated with 100 nM of phorbol 12-myristate 13-acetate (PMA, Sigma-Aldrich) for 24 h as described previously [18].

### AAA Mouse Models

The impact of genetic inhibition of EP4 on AAA formation was examined using the heterozygous EP4 knockout mouse (EP4^+/−^) since homozygous knockout is lethal [18]. AAA was induced by periaoritc application of 0.5 M CaCl_2_ as described previously [17]. The sham group received saline instead of CaCl_2_. Aortic morphometry was performed 4 weeks after CaCl_2_ treatment.

AAA was also induced after crossing EP4^+/−^ [18] with the apolipoprotein E knockout mouse (ApoE^−/−^) (The Jackson Laboratory, Bar Harbor, ME, USA). Briefly, EP4^−/−^ mice with a C57BL/6 genetic background [18] were crossed with ApoE^−/−^ mice with the same genetic background, and the resulting mice (EP4^+/−^/ApoE^+/−^) were intercrossed to generate EP4^+/−^/ApoE^−/−^ mice and their littermate controls (EP4^+/+^/ApoE^−/−^). To induce AAA formation, male EP4^+/−^/ApoE^−/−^ mice and littermate EP4^+/+^/ApoE^−/−^ mice were infused with AngII (1,000 ng/min/kg; Sigma-Aldrich) via an osmotic minipump (Alzet, model 2004, Cupertino, CA, USA) for 4 weeks, as described previously [19].

The effect of pharmacological inhibition of EP4 was examined in ApoE^−/−^ mice infused with AngII. Simultaneously, mice were orally administered ONO-AE3-208 (0.005, 0.01, 0.05, 0.5 mg/kg/day) as a bolus for 4 weeks. At the end of AngII infusion, the mice were sacrificed by an overdose of pentobarbital and were perfusion-fixed with a mixture of 3.7% formaldehyde in PBS at physiological perfusion pressure. Abdominal aorta were photographed to determine their external diameter, and also used for histological analyses. All aortic morphometries were performed by an investigator in a blinded manner. For gelatin zymography, we used freshly isolated aortic tissues at the end of AngII infusion.

### Ethics Statement

All protocols using human specimens were approved by the Institutional Review Board at Yokohama City University and all samples were obtained after receiving written informed consent. All animal studies were approved by the Institutional Animal Care and Use Committees of Yokohama City University.

### Quantitative Reverse Transcriptase-Polymerase Chain Reaction (RT-PCR)

Isolation of total RNA and generation of cDNA were performed and RT-PCR analysis was done as described previously [20]. The primers were designed based on rat nucleotide sequences of human EP1(NM_000955) (5′-GGA TGT ACA CCA AGG GTC CAG-3′ and 5′-TCA TGG TGG TGT CGT GCA TC-3′), human EP2 (NM_000956) (5′-AGG ACT GAA CGC ATT AGT CTC AGA A-3′ and 5′-CTC CTG GCT ATC ATG ACC ATC AC-3′), human EP3 variants 1–9,11(NR_028292-4, NM_198714-9, NM_001126044) (5′-GGA CTA GCT CTT CGG ATA ACT-3′ and 5′-GCA GTG CTC AAC TGA TGT CT-3′), human EP4 (NM_000958) (5′-AAC TTG ATG GCT GCG AAG ACC TAC-3′ and 5′-TTC TAA TAT CTG GGC CTC TGC TGT G-3′), and mouse EP4 (5′-TTC CCG CAG TGA TGT TCA TCT-3′ and 5′-CGA CTT GCA CAA TAC TAC GAT GG-3′). Each primer set was designed between multiple exons, and PCR products were confirmed by sequencing. The abundance of each gene was determined relative to the 18S transcript.

### Immunoblot Analysis

Proteins from whole cells were analyzed by immunoblotting as described previously [20].

### Tissue Staining and Immunohistochemistry

Elastic fiber formation was evaluated by elastica van Gieson staining. Immunohistochemical analysis was performed as described previously [20], [21]. A color extraction method using Keyence software was performed to quantify elastic fiber formation and expression of EP4.

### Gelatin Zymography

MMP activity was examined by gelatin zymography as described previously [17].

### ELISA

IL-6 and MCP-1 in conditioned media were measured using ELISA (R&D Systems, Minneapolis, MN, USA) according to the manufacturer's instructions.

### Statistical Analysis

Data are shown as the mean ± SEM of independent experiments. Unpaired Student's *t*-test, one-way ANOVA followed by Student-Newman-Keuls multiple comparison test, and Pearson's Correlation Coefficient were used to determine the statistical significance of the data. A value of *P*<0.05 was considered significant.

## Results

### Prostaglandin E Receptor EP4 Was Up-regulated in Aneurysmal Areas of Human Abdominal Aortas

**Figure 1:**
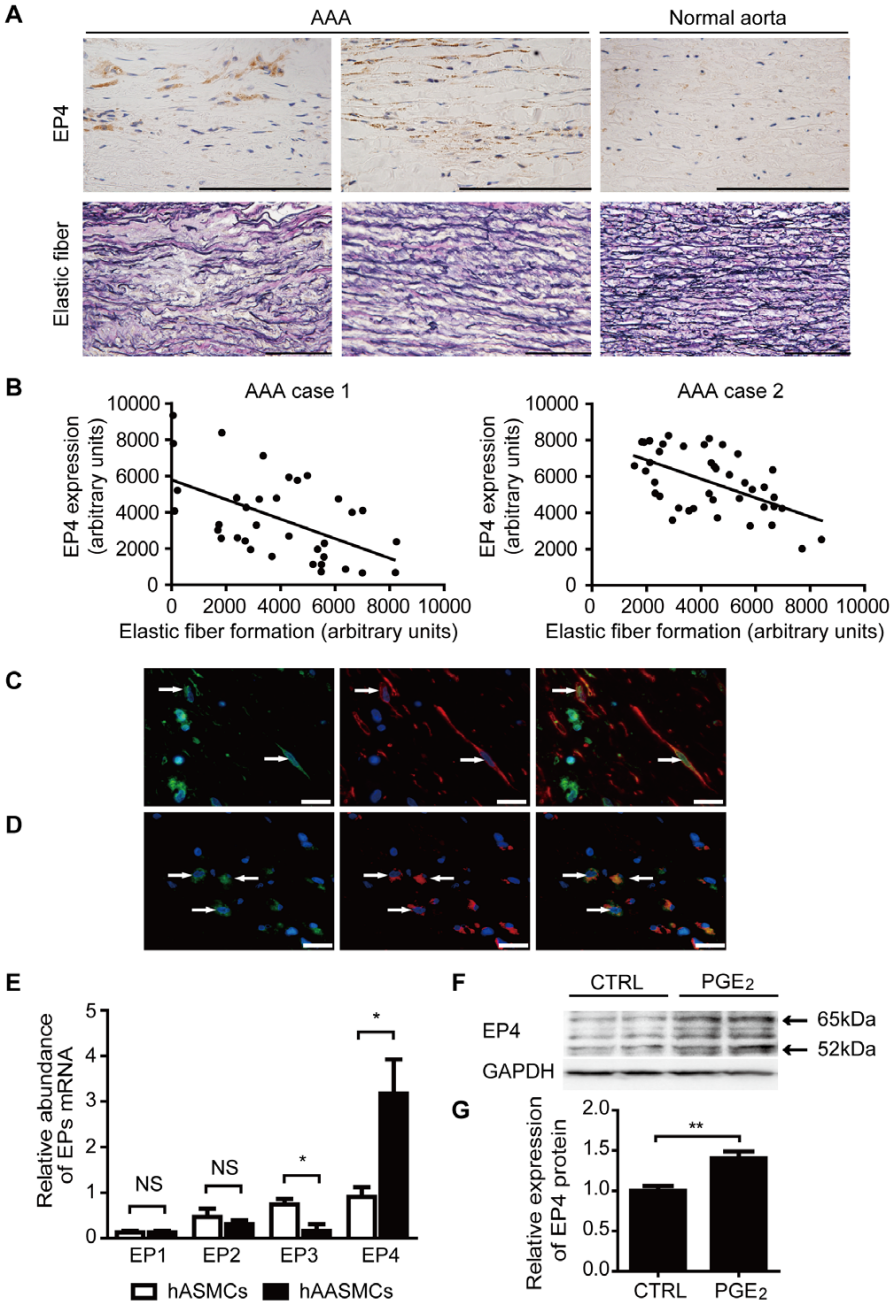
EP4 expression is increased in human AAA tissue. A, Immunohistochemistry for EP4 in human AAA tissues and aortic tissue from individuals who died of unrelated causes (upper panels). Brown areas indicate expression of EP4. Elastica van Gieson-stained aortic tissues (lower panels). Scale bars: 100 µm. B, Representative correlations between EP4 protein expression and elastic fiber formation in human AAA tissues. C, Immunofluorescent staining for EP4 (green, left panel) and α-smooth muscle actin (red, middle panel). Merged image is shown in the right panel. Arrows indicate EP4- and α-smooth muscle actin-positive cells. D, Immunofluorescent staining for EP4 (green, left panel) and CD68 (red, middle panel). Merged image is shown in the right panel. Arrows indicate EP4- and CD68-positive cells. Scale bars: 20 µm. E, Expression of EP1–4 using quantitative RT-PCR in hASMCs and hAASMCs. n = 5. F, Immunoblotting for EP4 and GAPDH in hASMCs incubated in the presence or absence of 1 µM of PGE_2_ for 72 h. G, Quantification of F. n = 4–5. *, *P*<0.05; **, *P*<0.01; NS, not significant.

In human tissue samples obtained from AAA surgeries, we found that EP4 expression and elastic fiber degradation were both enhanced in aneurysmal areas relative to that in normal areas. Indeed, statistical analysis revealed that the correlation was significant between the amount of EP4 expression and the degree of elastic fiber degradation (p<0.0001 to 0.0168) (**Figures 1A and B** **, and** **Table 1**).

**Table 1:** Correlation between elastic fiber formation and EP4 expression in AAA tissues.

	age	gender	r	number of sampling point	*P* value
1	76	M	−0.5386	35	0.0008***
2	63	M	−0.5645	41	0.0001***
3	76	M	−0.8000	25	<0.0001***
4	80	M	−0.4607	29	0.011*
5	70	M	−0.5454	39	0.0003***
6	76	M	−0.7571	60	<0.0001***
7	70	M	−0.4333	30	0.0168*
8	89	F	−0.5200	44	0.0003***

r: correlation coefficient; n: number of sampling points.

* , *P*<0.05;

** , *P*<0.01;

*** , *P*<0.001.

Previous studies have demonstrated that EP4 is abundantly expressed as primary PGE_2_ receptors in macrophages in aneurysmal areas [22]. However, whether or not other cell types such as ASMCs also express EP4 and other subtypes was not determined. We found, by immunohistochemistry of tissue samples, that EP4 was abundantly expressed in both α-smooth muscle actin-positive cells, i.e., ASMCs, (**Figure 1C**) and in CD68-positive cells, i.e., macrophages (**Figure 1D**). EP subtype expression was further characterized in cultured hAASMCs isolated from AAA tissue (**Figure 1E**). We found that EP4 mRNA expression was much greater than that of other EP subtypes such as EP1, EP2, and EP3. In contrast, when hASMCs isolated from normal aorta were examined, EP4 mRNA expression was not increased, suggesting that EP4 was increased only in aneurysmal ASMCs. When normal hASMCs were stimulated with PGE_2_, however, EP4 protein expression was significantly increased (**Figures 1F and G**). Thus, we can tentatively speculate that local production of PGE_2_ increased EP4 in the ASMCs in aneurysmal areas, which might play a role in AAA exacerbation.

### EP4 Stimulation Increased MMP-2 Activity and IL-6 Production in hAASMCs and Human AAA Tissue Organ Cultures

**Figure 2:**
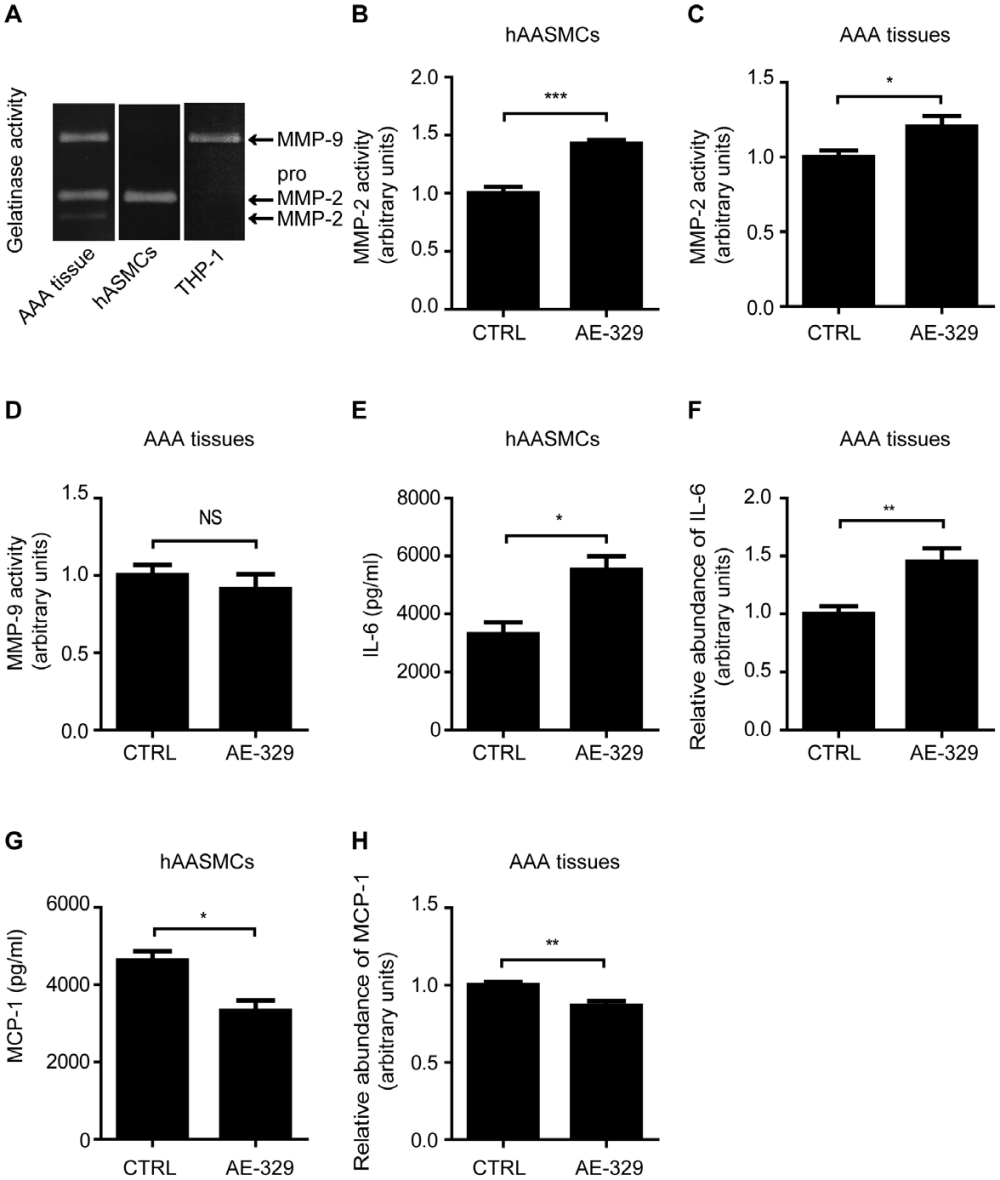
EP4 signaling increased MMP-2 activation and IL-6 production in hAASMCs and human AAA tissues. A, Representative images of gelatin zymography of human AAA tissue, hASMCs, and THP-1 treated with 100 nM of PMA. B, E and G, MMP-2 activation, IL-6, and MCP-1 production in supernatant of hAASMCs treated with or without 1 µM of ONO-AE1-329 (AE1–329) for 48 h, respectively. n = 5–7. C, D, F, and H, MMP-2 and MMP-9 activation, IL-6 and MCP-1 production in supernatant of human AAA tissue organ cultures incubated in the presence or absence of 1 µM of ONO-AE1-329 (AE1–329) for 48 h, respectively. n = 10–11. *, *P*<0.05; **, *P*<0.01; ***, *P*<0.001; NS, not significant.

Previous reports have demonstrated that MMP-2 and MMP-9, which are respectively derived from SMCs and macrophages, play important roles in the progression of aortic aneurysms [9]. We also found that MMP-2 and MMP-9 were both abundant in the supernatants of human AAA tissue organ cultures (**Figure 2A**). We also confirmed that MMP-2 was produced exclusively by hASMCs, and MMP-9 by THP-1 macrophage cells [9]. When hAASMCs or human AAA tissue organ cultures were stimulated with the EP4 agonist ONO-AE1-329, we found that MMP-2 activity was significantly increased in both preparations (**Figure 2B and C**). In contrast, EP4 stimulation did not alter MMP-9 activation in organ cultures (**Figure 2D**). We also examined the effect of EP4 stimulation on cytokines and chemokine because vascular inflammation is another prominent feature of atherosclerotic AAA [1]. We found that EP4 stimulation increased IL-6 production but decreased MCP-1 production in both hASMCs (**Figures 2E and G**) and human AAA tissue organ cultures (**Figures 2F and H**). These findings suggest that enhanced EP4 signaling may increase MMP activity and inflammatory response in AAA.

### Genetic Deletion of EP4 Reduced AAA Formation in vivo

Since the above experiments implied that EP4 stimulation has an exacerbating effect on AAA formation, we hypothesized that inhibition of EP4 signaling might have a salutary effect. We therefore examined the effect of genetic disruption of EP4 signaling by using EP4^+/−^ mice, because the total knockout of EP4 is lethal during the neonatal period [18]. EP4 expression in EP4^+/−^ mice was decreased to 43±6% (aorta) and 63±10% (heart), relative to that of wild-type mice (n = 6, *P*<0.05).

**Figure 3:**
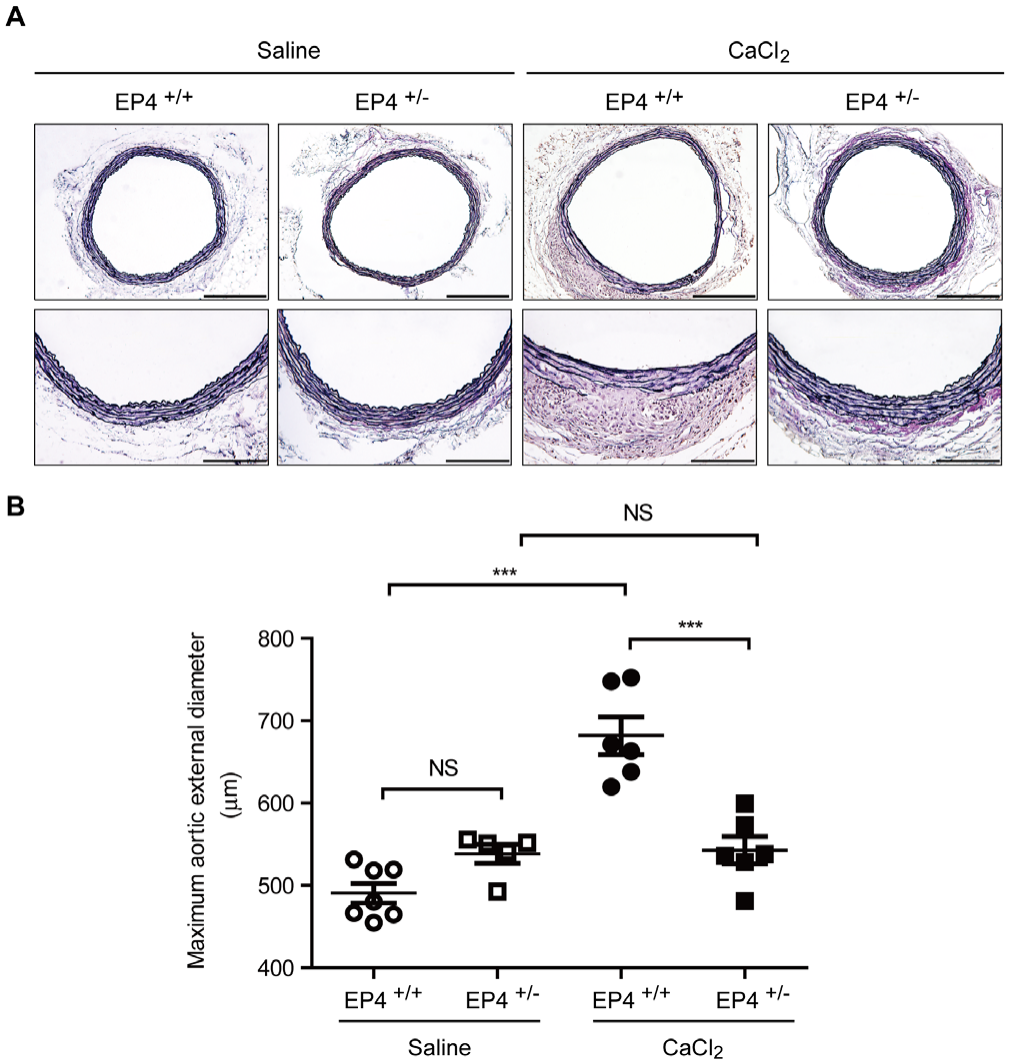
CaCl_2_-induced AAA formation is attenuated in EP4^+/−^ mice. A, Representative images of elastica van Gieson-stained tissue of EP4^+/−^ and EP4^+/+^ mice treated with saline or CaCl_2_. Lower panels (Scale bars: 100 µm) show higher magnification portions of upper panel images (Scale bars: 200 µm). B, Maximum aortic external diameter of AAA formation induced by CaCl_2_ in EP4^+/−^ and EP4^+/+^ mice treated with saline or CaCl_2_. n = 5–7. ***, *P*<0.001; NS, not significant.

**Figure 4:**
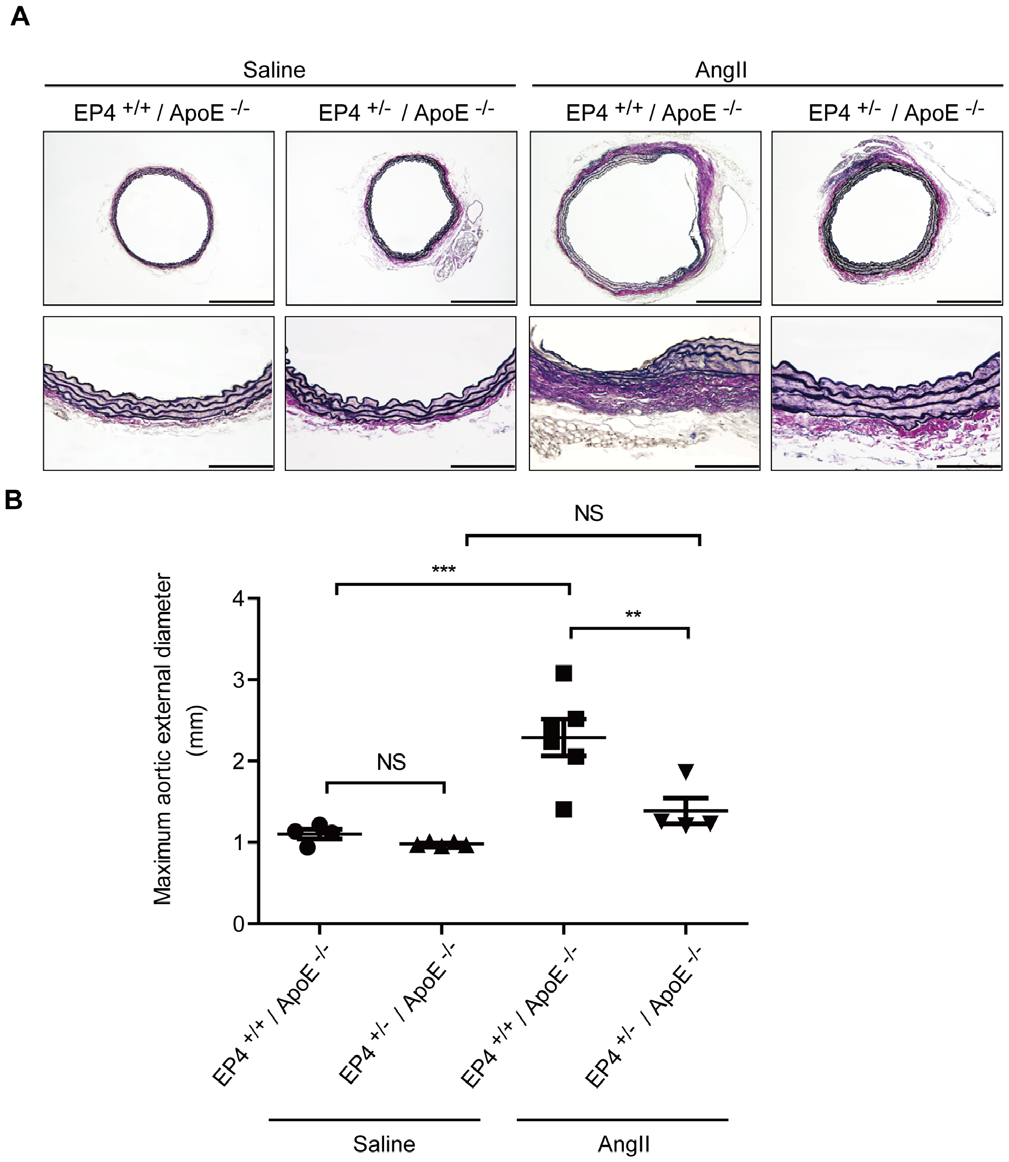
AngII-induced AAA formation is attenuated in EP4^+/−^/ApoE^−/−^ mice. A, Representative images of elastica van Gieson-stained tissue of EP4^+/−^/ApoE^−/−^ and EP4^+/+^/ApoE^−/−^ mice treated with saline or AngII. Lower panels (Scale bars: 100 µm) show higher magnification portions of upper panel images (Scale bars: 200 µm). B, Maximum aortic external diameter of AAA induced by AngII in EP4^+/−^/ApoE^−/−^ and EP4^+/+^/ApoE^−/−^ mice treated with saline or AngII. n = 4–6. **, *P*<0.01; ***, *P*<0.001; NS, not significant.

When CaCl_2_ was applied to the mouse abdominal aorta [17], aneurysmal formation with elastic fiber degradation was induced. However, these changes were significantly decreased in EP4^+/−^ mice (**Figures 3A and B**). In the absence of CaCl_2_ application, however, no significant difference between EP4^+/−^ and EP4^+/+^ mice was seen. Similarly, we examined AAA formation in EP4^+/−^ mice crossed with ApoE^−/−^ mice (EP4^+/+^/ApoE^−/−^), with AAA induced by continuous AngII infusion [19]. We found that the incidence of aortic aneurysm formation as well as elastic fiber degradation was significantly decreased in EP4^+/−^/ApoE^−/−^ mice (**Figures 4A and B**). In the absence of AngII infusion, however, no significant difference between EP4^+/−^/ApoE^−/−^ and EP4^+/+^/ApoE^−/−^ mice was observed. Thus, in two distinct models, EP4 deletion decreased AAA formation.

### EP4 Antagonist Reduced AAA Formation in vivo

**Figure 5:**
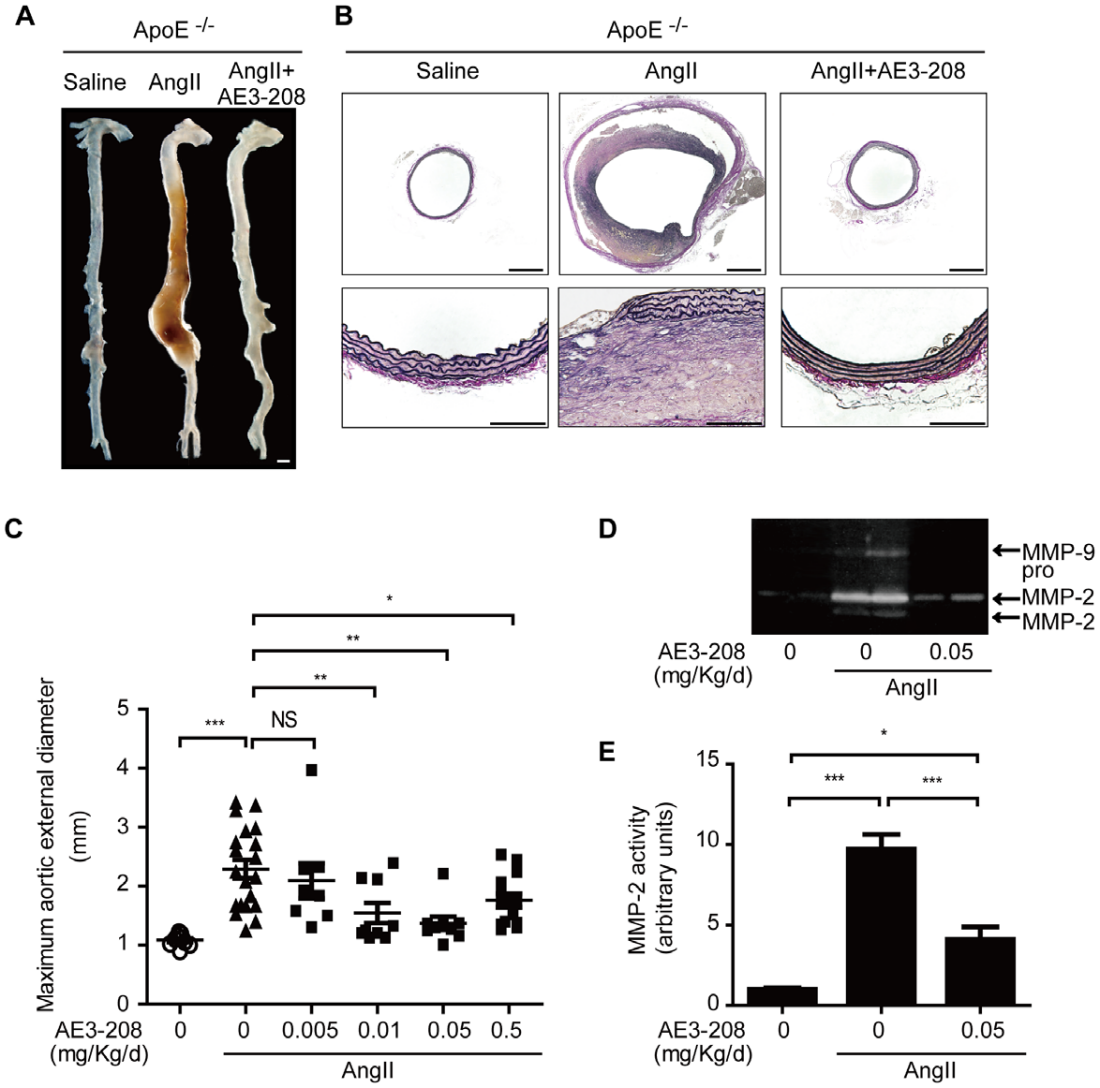
EP4 antagonist attenuated AngII-induced AAA formation in ApoE^−/−^ mice. A, Representative image of aorta of ApoE^−/−^ mice treated with saline, AngII, or AngII+ONO-AE3-208 (AE3–208) (0.05 mg/Kg/d). Scale bar: 1 mm. B, Elastica van Gieson–stained tissue of aortas shown in A. Lower panels (Scale bars: 100 µm) show higher magnification portions of upper panel images (Scale bars: 500 µm). C, Maximum aortic external diameter of AngII-induced AAA formation induced by AngII in ApoE^−/−^ mice treated with saline, AngII or AngII+ONO-AE3-208. n = 8–20. D, Representative images of gelatin zymography of AAA tissues of ApoE^−/−^ mice treated with saline, AngII, or AngII+ONO-AE3-208 (0.05 mg/Kg/d). E, Quantification of D. n = 8–12. *, *P*<0.05; **, *P*<0.01; ***, *P*<0.001; NS, not significant.

We also examined the effect of pharmacological inhibition of EP4 by ONO-AE3-208, an EP4 antagonist [23], with AAA formation induced by AngII infusion in ApoE^−/−^ mice. ONO-AE3-208 (0.005–0.05 mg/kg/day) was administered orally for 4 weeks. We found that elastic fiber degradation and thus AAA formation were inhibited by ONO-AE3-208 in a dose-dependent manner (**Figures 5A, B and C**). MMP-2 and MMP-9 activation were increased by AngII infusion, but activation was decreased in the presence of ONO-AE3-208 (0.05 mg/kg/day) (**Figures 5D and E**).

### EP4 Antagonist Inhibited MMP-2 Activation and IL-6 Production in Explants of Human AAA

**Figure 6:**
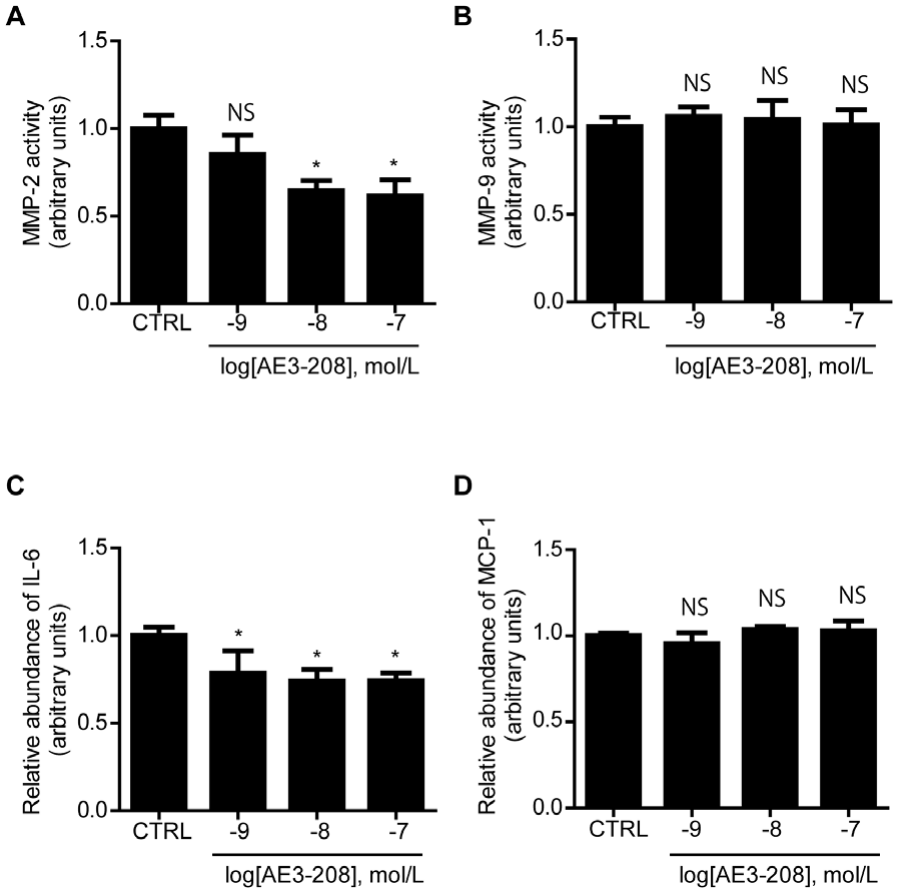
EP4 antagonist attenuated MMP-2 activation and IL-6 production in human AAA tissues. A, MMP-2 activity, B, MMP-9 activity, C, IL-6 production, and D, MCP-1 production. Supernatants of human AAA tissue organ cultures incubated in the presence or absence of and increasing concentrations of ONO-AE3-208 (AE3–208). n = 10–20. *, *P*<0.05 vs. control (CTRL); NS, not significant.

We further examined the effect of the EP4 antagonist on cytokine and chemokine production in human AAA tissues. ONO-AE3-208 significantly decreased MMP-2 activation in a dose-dependent manner (10^−8^ M to 10^−7^ M) (**Figure 6A**), which was most likely related to ASMCs. MMP-9 activation was unaltered, which was most likely related to macrophages (**Figure 6B**). IL-6 production was decreased in a dose-dependent manner at dosages between 10^−9^ M and 10^−7^ M (**Figure 6C**), but MCP-1 production was unchanged (**Figure 6D**).

## Discussion

Our study demonstrated that EP4 expression was increased in the aneurysmal areas of human AAA tissues, both in ASMCs as well as in macrophages in the lesion. Importantly, EP4 expression was not increased in normal human ASMCs, but was induced when normal cells were stimulated by PGE_2_. When EP4 was stimulated in hAASMCs and AAA tissue organ cultures, both MMP-2 activity and IL-6 production were increased. With these findings in mind, we examined the effect of EP4 inhibition, either by EP4 gene disruption (EP4^+/−^) or the use of an EP4 antagonist (ONO-AE3-208). In various models of AAA, induced by CaCl_2_ or AngII infusion in ApoE^−/−^ mice, EP4 inhibition significantly decreased AAA formation. Furthermore, the EP4 antagonist inhibited IL-6 production and MMP-2 activation in human AAA tissues, suggesting a mechanism for EP4 antagonist-mediated inhibition of AAA formation. Accordingly, we propose that EP4 inhibition may serve as an effective pharmacological therapy to prevent the exacerbation of AAA in humans.

Many molecules have been explored as potential targets for a pharmacological therapy of AAA. TGFβ and AngII, for example, are well known to be increased in AAA. However, it remains controversial whether pharmacological inhibition of these signals can provide effective therapy in AAA [24]. Because it is also well known that COX-2-dependent PGE_2_ synthesis is increased, leading to exacerbation of AAA, we hypothesized that this may serve as a possible target for pharmacotherapy as well. Indeed, a previous study demonstrated that COX-2 inhibition by non-steroidal anti-inflammatory drugs prevented AAA exacerbation [5]. Similarly, Gitlin *et al.* showed that COX-2 deficient mice exhibited decreased AngII-induced AAA formation [14]. These findings are in agreement with the fact that PGE_2_ is synthesized via COX-2 at high concentration in AAA walls [5], [10], so inhibiting it may impede AAA exacerbation.

Because recent clinical studies have shown that COX-2 inhibition *per se* can induce multiple cardiovascular adverse events [15], [16], we aimed in this study to inhibit processes further downstream from the COX-2/PGE_2_ signal. For PGE_2_, there are four receptor subtypes: EP1, EP2, EP3, and EP4 [25]. EP4 is dominantly expressed in macrophages [26], and is a major stimulator of cytokines and proteolytic enzymes production such as MMPs. EP4 is therefore importantly involved in AAA pathophysiology, and many studies have demonstrated that EP4 signaling increases MMP-9 activation in macrophages [27], [28], [29], leading to exacerbation of AAA [9]. Thus, inhibition of EP4, particularly in macrophages, may be of benefit in preventing AAA. Unexpectedly, however, a very recent study demonstrated that EP4 disruption in bone marrow-derived cells augmented elastin fragmentation and exacerbated AAA formation [30]. Possible reasons for this unfavorable finding may include that EP4 disruption increased MCP-1 because EP4 stimulation can inhibit MCP-1 production in macrophages [31], [32]. Consequently, macrophage-selective inhibition of EP4 may not provide an effective therapy for AAA.

Our study, in contrast, demonstrated the effectiveness of systemic administration of an EP4 antagonist, which inhibits the EP4 signal in all cell types, particularly those with high EP4 expression. Importantly, our study demonstrated, for the first time, that normal ASMCs can increase EP4 expression when stimulated by PGE_2_. Thus, inflammation in AAA lesions may have increased EP4 expression in ASMCs. The effectiveness of EP4 signaling inhibition in ameliorating AAA exacerbation is also supported by other findings in this study. EP4 stimulation increased IL-6 production and MMP-2 activation in ASMCs, and the use of an EP4 antagonist inhibited IL-6 production and MMP-2 activation in human AAA tissue organ cultures. Although it is known that MMP-2 is mainly expressed in hASMCs [9], PGE_2_-mediated regulation of MMP-2 has not been demonstrated previously. Here, we demonstrated that EP4 is a potent regulator of MMP-2 in ASMCs and that this regulation can be indirectly enhanced by IL-6. Our study also indicated that EP4 signaling is a potent inducer of IL-6 production in ASMCs. Because IL-6 *per se* can increase MMP-2 production [33], an EP4 antagonist might indirectly inhibit MMP-2 production by regulating IL-6 in ASMCs as well.

From the view point of pharmacological therapy, when 10 mg/kg/day of ONO-AE3-208 was administered orally as a bolus, the peak plasma concentration was 677 ng/ml (1.7 µM) after 0.25 hours, as shown in a previous study describing a different use [23]. Accordingly, when 0.01 mg/kg/day of ONO-AE3-208 was orally administered in our study, the peak expected plasma concentration in mice was approximately 1.7 nM. Since the Ki value of ONO-AE3-208 was 1.3, 30, 790, and 2,400 nM for EP4, EP3, FP, and TP, respectively [23], our dosages of the EP4 antagonist are likely to have inhibited EP4 in a selective manner. Indeed, this EP4 antagonist was effective in 0.01–0.5 mg/kg/day in our mouse study.

In conclusion, this study demonstrated that selective EP4 inhibition was efficacious in inhibiting the exacerbation of AAA formation in a number of mouse models. In particular, pharmacological inhibition of EP4 signaling by an EP4 antagonist was effective at relatively low doses. Although we have not examined the effect of EP4 inhibition on other tissues or organs that also express high EP4, our study suggests, at the very least, that pharmacological EP4 inhibition may serve as a new therapeutic strategy for aneurysmal diseases for which effective medical therapy is currently unavailable.
